# Identification of Genes Related to Rapid Growth of Giant Grouper (*Epinephelus lanceolatus*) Based on Self-Cross Population of Hulong Hybrid Grouper (*E. fuscoguttatus* ♀ × *E. lanceolatus* ♂)

**DOI:** 10.3390/ani15243599

**Published:** 2025-12-15

**Authors:** Leilei Zeng, Tong Wang, Qichuang Wei, Yuhao Tao, Leyi Chang, Yanzhao Zhao, Xunran Pan, Yingjie Li, Zining Meng, Yang Yang, Xiaochun Liu

**Affiliations:** 1 State Key Laboratory of Biocontrol/Southern Marine Science and Engineering Guangdong Laboratory (Zhuhai), Institute of Aquatic Economic Animals and Guangdong Provincial Key Laboratory of Aquatic Economic Animals, School of Life Sciences, Sun Yat-Sen University, Guangzhou 510275, China; 2 China-ASEAN Belt and Road Joint Laboratory on Mariculture Technology, Guangzhou 510275, China; 3 Key Laboratory of Tropical Marine Fish Germplasm Innovation and Utilization, Ministry of Agriculture and Rural Affairs, Hainan Engineering Research Center for Germplasm Innovation and Utilization, Hainan Chenhai Aquatic Co., Ltd., Sanya 572024, China

**Keywords:** giant grouper, growth, SNP, hybrid, hulong grouper

## Abstract

Growth is a key genetic improvement target in aquaculture. The giant grouper (*Epinephelus lanceolatus*), the largest and fastest-growing grouper species, is an important aquaculture species and also an ideal male parent in grouper hybrid breeding, such as hulong hybrid grouper (*E. fuscoguttatus* ♀ × *E. lanceolatus* ♂). However, the genetic basis of this rapid growth is unclear. In this study, we established a hulong hybrid grouper self-cross population, observing significant growth segregation. Using Bulked Segregant Analysis (BSA) and RNA-seq on extreme growth groups, we identified a significant growth-related quantitative trait locus (QTL) on chromosome 2, containing 23 candidate genes and 5 growth-correlated non-synonymous SNPs. Transcriptome analysis revealed 4074 differentially expressed genes. Integrating these results, we identified three critical genes: *iqgap1*, *mex3b*, and *ndufs3*, involved in cell proliferation, embryonic development, and energy metabolism, respectively. Their expression patterns further supported their association with giant grouper’s rapid growth. Our findings provide crucial insights into giant grouper growth mechanisms and valuable molecular markers for grouper breeding.

## Simple Summary

Giant grouper (*Epinephelus lanceolatus*) is an important aquaculture species and also an ideal male parent in grouper hybrid breeding for their fast growth. However, the genetic basis of this rapid growth is unclear. We established an F_2_ population using the giant grouper as the paternal parent and found significant differences in individual growth rates. By BSA (Bulked Segregant Analysis) and RNA-seq analysis on extreme growth groups, a specific region on chromosome 2 related to growth was identified. Within this region, we identified five key genetic variations and three important genes (*iqgap1*, *mex3b*, and *ndufs3*) that play roles in cell growth, development, and energy use. These genes show different expression levels in giant grouper compared to other groupers, highlighting their connection to rapid growth. This discovery helps us understand giant grouper’s growth advantage and provides valuable tools for breeding faster-growing fish for aquaculture.

## 1. Introduction

Growth is one of the most economically important characteristics and a primary target trait for genetic improvement in aquaculture species [1]. Accelerating the growth rate of fish can significantly shorten the culture period, reduce production costs, mitigate farming risks, and increase profitability. Understanding of the genetic basis and regulatory mechanisms underlying growth is essential for the genetic improvement in cultured fish. Growth is a complex quantitative trait [2], which is regulated by the Growth Hormone-Insulin-like Growth Factor (GH-IGF) axis [3]. Recently, an increasing number of other growth-related genes have been identified as being involved in a variety of biological processes, including immune response, feeding regulation, metabolism, and bone mineralization [4,5,6,7]. The genetic basis of growth regulation has not been fully elucidated.

A large number of studies have been conducted on the genetic mechanisms of economic traits, achieving significant progress in aquatic species using various effective analytical methods. For instance, a few potential QTLs associated with the omega-3 fatty acid composition of Atlantic salmon fillets were identified through a genome-wide association study (GWAS) [8], a quantitative trait locus (QTL) associated with rainbow trout resistance to viral hemorrhagic septicemia virus was found by QTL mapping [9], and a growth-related QTL was discovered in bighead carp by bulked segregant analysis (BSA) [10]. However, the identified genomic regions are often excessively broad and contain hundreds of genes due to the limited mapping resolution of the experimental populations, which makes it extremely difficult to distinguish the true causal gene from the numerous candidate genes. Furthermore, the complexity of phenotypic variation, which is frequently shaped by gene interactions (epistasis) and environmental factors, adds to the challenge of pinpointing specific causal variants [11,12]. Therefore, refining these broad regions through fine-mapping or the use of specific segregating populations remains a critical step for precise marker identification.

Hybridization is a crucial breeding technique. Combining it with different breeding schemes can obtain ideal populations for genetic studies, such as backcross (BC) populations, F_2_ segregating populations, and multi-parent advanced generation inter-cross (MAGIC) populations. Among these populations, the self-crossing population of hybrid F1 (i.e., an F_2_ segregating population) is considered an excellent resource for trait analysis and marker identification [13]. During the generation of the F_2_ population, homologous chromosomes randomly separate into gametes and subsequent recombination events generate abundant genetic variations and segregation of characters [14]. Compared to pure-line self-crossing populations or backcrossing populations, the self-crossing population of hybrid F1 has superior power of genetic mapping [15]. Consequently, the F_2_ segregating populations have been widely applied to identify trait-associated molecular markers and elucidate genetic mechanisms in both animals and plants. For example, two key genes affecting average daily gain and backfat thickness were identified in an F_2_ population of Duroc × Pietrain intercross [16], an F_2_ population of a mallard × Pekin duck cross was used to map major genes for body size and plumage color [17], and an F_2_ population of the yellow river carp was utilized to identify five significant growth-related genes [18].

Groupers are a group of economically significant and high-value marine fish. In recent years, the grouper aquaculture industry has developed rapidly [19]. As the largest and fastest-growing grouper, the giant grouper can grow up to 2.7 m and 400 kg [20]. It is not only a cultured species, but also an ideal male parent in grouper hybrid breeding, such as *E. moara* ♀ × *E. lanceolatus* ♂ [21], *E. fuscoguttatus* ♀ × *E. lanceolatus* ♂ [22], and *Cromileptes altivelis* ♀ × *E. lanceolatus* ♂ [23]. The growth rate of these hybrid groupers has been significantly improved. Among the hybrid groupers, *E. fuscoguttatus* ♀ × *E. lanceolatus* ♂, called hulong hybrid grouper in China, has excellent growth performance, probably due to inheriting genes of rapid growth from the giant grouper, and it has become the primary cultured grouper species. Although some growth-related candidate genes have been identified through GWAS and QTL-mapping based on the giant grouper pure-line self-crossing populations [24,25], these candidate genes are minor-effect genes and the genetic basis of rapid growth in giant grouper is still largely unknown.

In this study, we successfully constructed a self-cross population of hulong hybrid grouper and selected two groups with extreme segregated body weight to investigate the genetic difference of growth. Whole-genome resequencing was performed on two pools with extreme segregated body weight, and BSA was implemented to map the associated QTLs and SNPs. On the other hand, transcriptomes were used to analyze the differentially expressed genes between the fast-growing group and the slow-growing group. Then, based on the results of BSA and transcriptome analysis, overlapping genes were selected as the key genes associated with rapid growth in giant grouper. Our findings provide novel insights into the genetic mechanisms regulating growth in giant grouper and molecular markers for marker-assisted breeding of grouper.

## 2. Materials and Methods

### 2.1. Experimental Animals

One male giant grouper and one female brown-marbled grouper were selected for crossbreeding to produce a full-sibling family of hulong hybrid grouper at Hainan Chenhai Aquatic Co. (Sanya, Hainan, China). Six sexually mature female and six male hulong hybrid groupers were cultured intensively to induce natural spawning and fertilization. Fertilized eggs were collected and transported to the Guangdong Marine Fisheries Experimental Center for incubation (Huizhou, Guangdong, China). Hatched larvae were reared in a recirculating aquaculture system (RAS) with a tank diameter of 5 m and a water depth of 1.2 m. Water temperature was maintained at 25–30 °C, and a natural photoperiod was applied. The previous month, rotifers were used as the initial feeding material. Then the juvenile fish were fed with food for fish larvae (Guangdong Yuequn Ocean Biotechnology Co., Ltd., Guangzhou, China) twice daily.

### 2.2. Measurement of Growth Phenotype and Sample Collection

At three months of age, the growth phenotypes of 1200 individuals from this self-cross population were randomly measured, including body weight, body length, total length, body width, and body height. Fin clips were collected and preserved in absolute ethanol for subsequent DNA extraction. Fish were anesthetized using MS-222 (100 mg/L), and muscle tissues from the four heaviest and four lightest individuals were collected. Similarly, muscle tissues were collected from three individuals of each of the brown-marbled grouper, giant grouper, and hulong hybrid grouper of the same weight from Hainan Chenhai Aquaculture Co., Ltd. (Sanya, China) for subsequent verification. The collected muscle tissues were immediately preserved in RNA keeper (Vazyme Biotech Co., Ltd., Nanjing, China) and stored at −80 °C for subsequent RNA extraction.

### 2.3. DNA and RNA Extraction

From a population of 1200 caudal fin samples, two extreme groups were constructed based on body weight: a fast-growing group comprising the 60 heaviest individuals and a slow-growing group comprising the 60 lightest individuals. Genomic DNA was extracted from these 120 individuals and their maternal brown-marbled grouper (F), using the Marine Animal DNA Extraction Kit (TIANGEN, Beijing, China). The purity of the extracted DNA was assessed using NanoDrop 2000 (Thermo Fisher, Waltham, MA, USA), and the integrity was evaluated by agarose gel electrophoresis. Finally, DNA concentration was precisely quantified using a Qubit fluorometer.

Total RNA was extracted from each sample from the fast-growing groups and slow-growing groups, as well as from similarly weighed brown-marbled grouper, giant grouper and hulong hybrid using TRIzol reagent (Invitrogen, Carlsbad, CA, USA). Following assessment of RNA integrity by 1% agarose gel electrophoresis, genomic DNA was removed with DNase I (Takara, Kyoto, Japan). The concentration of total RNA from each sample was quantified using a NanoDrop 2000 nucleic acid analyzer (Thermo Fisher, Waltham, MA, USA), and its purity was confirmed (OD260/280 between 1.9 and 2.0, indicating acceptable RNA purity).

### 2.4. Library Construction and Sequencing

DNA was subjected to quality control. Qualified DNA was randomly sheared into fragments of approximately 350 bp using a Covaris ultrasonicator (Covaris, Woburn, MA, USA). Sequencing libraries were then constructed via end-repair, A-tailing, adapter ligation, purification, and PCR amplification. The qualified libraries were sequenced on the BGI DNBSEQ-T7 platform using the PE150 mode to generate resequencing data.

Total RNA from both the fast-growing (FG) and slow-growing (SG) groups was randomly fragmented. The first strand of cDNA was synthesized by reverse transcription using random hexamers. The second strand of cDNA was then synthesized by adding buffer, dNTPs, RNase H, and DNA polymerase I. The resulting double-stranded cDNA was purified using AMPure XP beads. Following purification, the cDNA was subjected to end-repair, A-tailing, and sequencing adapter ligation. Fragments of approximately 150 bp were selected, and cDNA libraries were constructed through PCR enrichment. After quality control, the libraries were subjected to high-throughput sequencing to obtain RNA sequencing (RNA-Seq) data in PE150 mode.

### 2.5. SNP Detection and Genotyping

Raw sequencing reads were first subjected to quality control using fastp (v0.23.2) [26] with default parameters to remove adapter sequences and low-quality reads. The resulting high-quality, clean reads from each sample were then aligned to the reference genome (NCBI GenBank: GCF_011397635.1) using the mem algorithm in BWA (v0.7.17) [27]. The output BAM files were sorted by coordinate, and potential PCR duplicates were removed using the Picard. Next, an index was created for the sorted BAM files using SAMtools (v1.13) [28]. SNPs were then detected with GATK4 (v4.1.7.0) [29], yielding a VCF file. The SNPs were filtered with VCFtools [30] according to the following standards: a minor allele frequency of less than 10% (--max-maf 0.1); a maximum missing rate of 1 (--max-missing 1); retention of only SNPs (--keep-only-snp); and a minimum depth of 5 (--minDP 5). High-quality SNPs were obtained after filtering.

### 2.6. BSA-seq Analysis and Candidate Gene Annotation

Based on the high-quality SNPs, the qtlseq software (v2.2.4) [31] was used to identify regions associated with growth traits. The analysis was conducted with the following parameters: a window size of 2000 kb (--window-size 2000), a step size of 100 kb (--window-step 100), and the exclusion of markers with an SNP index below 0.3 (--min-SNP index 0.3). All other parameters were set to their default values. Genomic regions where the ΔSNP-index exceeded the 95% confidence interval were identified as candidate quantitative trait loci (QTLs). The statistical G′ values of each SNP within a 2000 kb sliding window are calculated using the software QTLseqr (v0.7.0) [32] to identify genomic regions where G′ statistic peaks occur, indicating the potential existence of QTLs. The q value < 0.05 is employed as the significance threshold for the G′ statistic. The regions where the ΔSNP-index exceeds 95% confidence interval and the q value of the G′ statistic is less than 0.05 are regarded as candidate QTL. Genes located within these candidate regions were extracted based on the reference genome’s GTF annotation file. Finally, all genes within the candidate regions were annotated using BLAST (v2.14.0).

### 2.7. Validation of the Growth-Related SNPs

Within the candidate regions identified through the ΔSNP-index analysis, SNP genotype information of each individual with extreme phenotypes was extracted from the resequencing data. Subsequently, for these candidate SNPs, the chi-square test was used to compare the frequency distributions of different genotypes or alleles in the fast-growing group and the slow-growing group to identify candidate SNPs. To screen the SNPs associated with growth, we conducted resequencing on two additional extreme groups. We selected two additional extreme populations from self-cross population of hulong hybrid grouper for resequencing. Based on the genotypes of each sample, we conducted a correlation analysis between the candidate SNPs and growth traits using the chi-square test.

### 2.8. RNA-seq Analysis

Raw RNA sequencing reads were first processed for quality control using fastp (v0.20.0) with default parameters to remove adapters and low-quality sequences. The resulting clean reads were then aligned to the reference genome (NCBI GenBank: GCF_011397635.1) using HISAT2 (v2.1.0) [33]. The SAM files generated by HISAT2 were subsequently converted to the binary format (BAM), sorted by coordinate, and indexed using SAMtools (v1.13). Gene expression levels were assessed based on fragments per kilobase of transcripts per million fragments mapped (FPKM) using the featureCounts program from the Subread package (v2.0.1) [34]. DESeq2 [35] was used for differentially expressed gene (DEGs) analysis. Genes with a *p*-value < 0.05 and an absolute log2-fold change of 1 or greater were considered differentially expressed genes.

### 2.9. Integrated BSA-seq and RNA-seq Analyses

Integrate the candidate QTL regions identified in the BSA-seq analysis with the differentially expressed genes (DEGs) obtained in the RNA-seq analysis. Genes with overlapping results from the BSA-seq analysis and RNA-seq analysis were used as key genes for subsequent validation.

### 2.10. Quantitative Real-Time PCR (qRT-PCR) Validation

A total of 1 μg of RNA per sample was reverse transcribed using the RevertAid First Strand cDNA Synthesis Kit (Invitrogen, Carlsbad, CA, USA). according to the manufacturer’s protocol. Primers were designed using Primer Premier 5.0 based on the reference genome (NCBI GenBank: GCF_011397635.1) in our study and synthesized by Sangon Biotech Co., Ltd. (Shanghai, China). Standard curve analysis was used to perform 10-fold serial dilutions of the cDNA template (5 gradients: 100, 10^−1^, 10^−2^, 10^−3^, 10^−4^) to verify the efficiency of the primers. The efficiency values of all primers were 95% to 100%, confirming the specificity and effectiveness of the amplification. A 10 μL reaction mixture contained 5.0 μL of SYBR Green PCR Master Mix (Applied Biosystems, Foster City, CA, USA), 0.4 μL of the forward and reverse primer mixture (2.5 μmol/L each), 0.4 μL of cDNA template, and 4.2 μL of nuclease-free water was amplified using the LightCycler^®^ 480 II Real-Time PCR System (Roche Diagnostics, Indianapolis, IN, USA). The thermal cycling conditions were as follows: an initial denaturation at 95 °C for 30 s, followed by 40 cycles of denaturation at 95 °C for 10 s and annealing/extension at 60 °C for 20 s. The relative expression levels of the key genes were calculated using the 2^−ΔΔCt^ method [36], with β-actin serving as the internal reference gene. And all samples were analyzed in technical triplicate. The primer sequences are listed in Table S1. Statistical analysis of gene expression differences between groups for the same tissue was performed using one-way analysis of variance (ANOVA) in GraphPad Prism (v10.1.2).

## 3. Results

### 3.1. Phenotypic Statistical Analysis of Growth Traits

**Figure 1:**
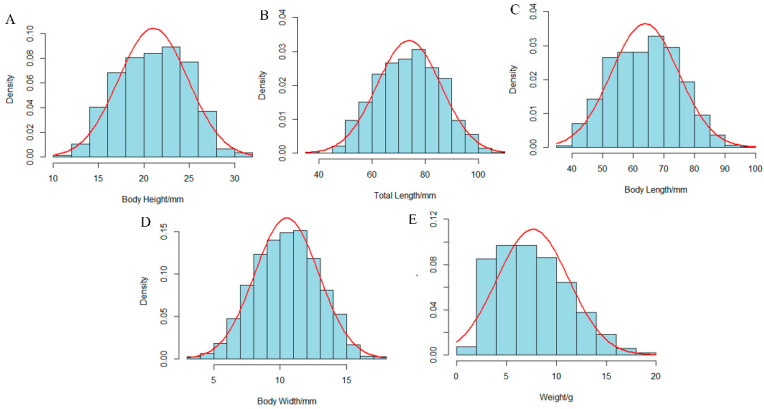
Frequency distribution histograms of five growth-related traits. The panels display the distributions for: (**A**) Body Height, (**B**) Total Length, (**C**) Body Length, (**D**) Body Width, and (**E**) Weight. The blue bars represent the observed frequency density of the phenotypic data, and the red curves represent the fitted normal distribution.

The descriptive statistics for five growth-related traits—body weight (W), body length (BL), total length (TL), body height (BH), and body width (BW)—were measured in 1200 individuals from the self-cross population of hulong hybrid grouper (Table 1 and Table 2). The mean values for W, BL, TL, BH, and BW were 7.72 ± 3.73 g, 63.93 ± 10.98 mm, 73.92 ± 12.18 mm, 21.07 ± 3.87 mm, and 10.51 ± 2.41 mm, respectively. The population showed a significant segregation of growth, with a minimum body weight of 1.30 g (Figure S1), a maximum body weight of 28.16 g and a coefficient of variation (CV) of 48.32%. In contrast, body length showed the lowest CV (17.18%). The frequency distribution histograms for all traits approximated a normal distribution (Figure 1). This was further supported by the Quantile-Quantile (QQ) plots (Supplementary Figure S2), where the majority of the data points closely followed the reference line, suggesting that the phenotypic data for all growth traits were approximately normally distributed.

### 3.2. Phenotypic Differentiation and Sequencing Data from Extreme Group

The significant segregation of character was observed between the fast-growing and slow-growing groups (Table 3). The maximum weight of the fast-growing group was 28.16 g, the minimum weight was 11.61 g, and the average weight was 16.14 ± 2.56 g. The maximum weight of the slow-growing group was 7.97 g, the minimum weight was 1.31 g, and the average weight was 2.56 ± 1.30 g. The mean body length of the fast-growing group (84.87 ± 3.92 mm) was nearly double that of the slow-growing group (45.05 ± 5.87 mm).

Whole-genome resequencing of the two groups with extreme segregated body weight generated a total of 1.40 Tb of raw data. After quality control and filtering, 1.22 Tb of high-quality clean data was retained for subsequent analysis. The clean reads from all individuals within each group were pooled to create a fast-growing and a slow-growing group, respectively. The effective sequencing depth for the fast-growing and slow-growing groups reached 813.55× and 873.71×, respectively. The quality of the sequencing data was high, with Q30 scores of 95.82% and 96.10%, and GC contents of 41.39% and 41.34% for the respective groups. Detailed statistics, including the number of clean reads for each pool, are provided in Supplementary Table S2.

### 3.3. Identification of Growth-Related QTLs

After alignment and variant calling, a total of 4,225,629 high-quality SNPs were retained for analysis following stringent filtering. These SNPs were distributed relatively evenly across the genome, with the number of SNPs per chromosome ranging from 100,160 to 206,940 (Figure S3).

**Figure 2:**
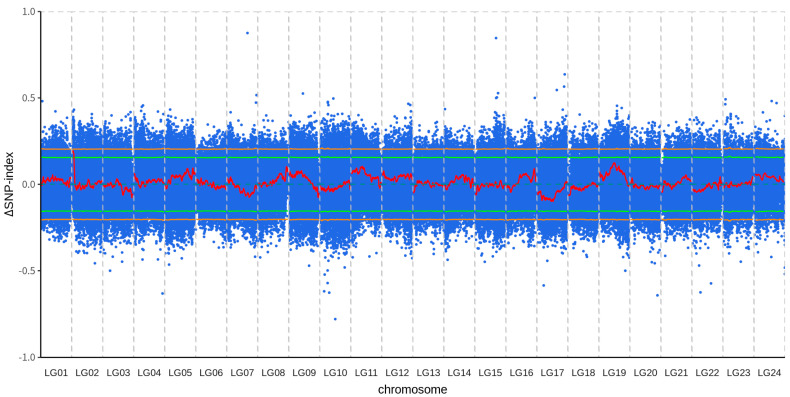
Genome-wide Δ(SNP−index) plot for the identification of QTLs associated with growth traits. Blue dots represent the Δ(SNP−index) value calculated for each sliding window. The red line represents the fitted Δ(SNP−index) value generated using a sliding window approach. The green and orange horizontal lines indicate the 95% and 99% confidence intervals, respectively, determined by permutation tests.

The BSA analysis results show that a QTL exceeds the 95% confidence interval, ranging from 1 Mb to 3.5 Mb on chromosome 2 (Figure 2; Supplementary Figure S4). The G′ statistic results also showed that there was a peak exceeding the threshold on chromosome 2 (G′ value > 16.44), ranging from 0.063 Mb bp to 3.1 Mb bp on chromosome 2 (Supplementary Figure S5). Therefore, a region from 1 Mb to 3.1 Mb on chromosome 2 was identified as a candidate QTL. This candidate QTL contained 23 annotated genes (Table 4) and a total of 3446 SNPs. 82 SNPs were located within the exonic regions of genes, comprising 31 non-synonymous and 51 synonymous mutations.

### 3.4. Validation of the Growth-Related SNPs

As a result, seven candidate SNPs were identified within the candidate QTL region (Supplementary Table S3). To screen the SNPs associated with growth, two other extreme groups (41 fast-growing individuals and 37 slow-growing individuals) were selected from a self-cross population of hulong hybrid grouper as the validation population. Then, genotyping of candidate SNPs in the validation population and correlation analysis between candidate SNPs and growth traits. Finally, the result showed that five SNPs were significantly associated with growth traits (Table 5).

### 3.5. Differential Gene Expression and Functional Enrichment Analysis

A total of 53.11 Gb of high-quality clean data was obtained from the transcriptome sequencing of eight samples. The quality of the sequencing data was good, with Q20 and Q30 scores exceeding 97.51% and 93.12%, respectively. The GC content ranged from 50.19% to 50.97%, and the average mapping rate of reads to the reference genome was over 82.24% (Supplementary Table S4). Comparative transcriptome analysis between the fast-growing FG and SG groups identified a total of 4074 differentially expressed genes (DEGs). Among these, 2143 genes were significantly up-regulated, while 1931 were down-regulated in the FG group relative to the SG group.

**Figure 3:**
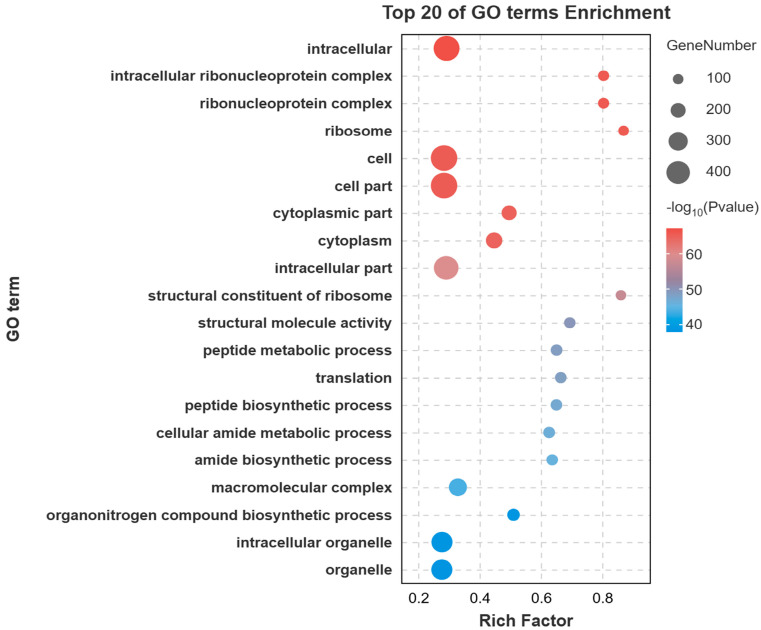
Top 20 GO terms for the DEGs. Bubble chart of the top enriched categories. The color scale indicates the significance level (e.g., *p*-value), while the bubble size corresponds to the number of enriched genes.

**Figure 4:**
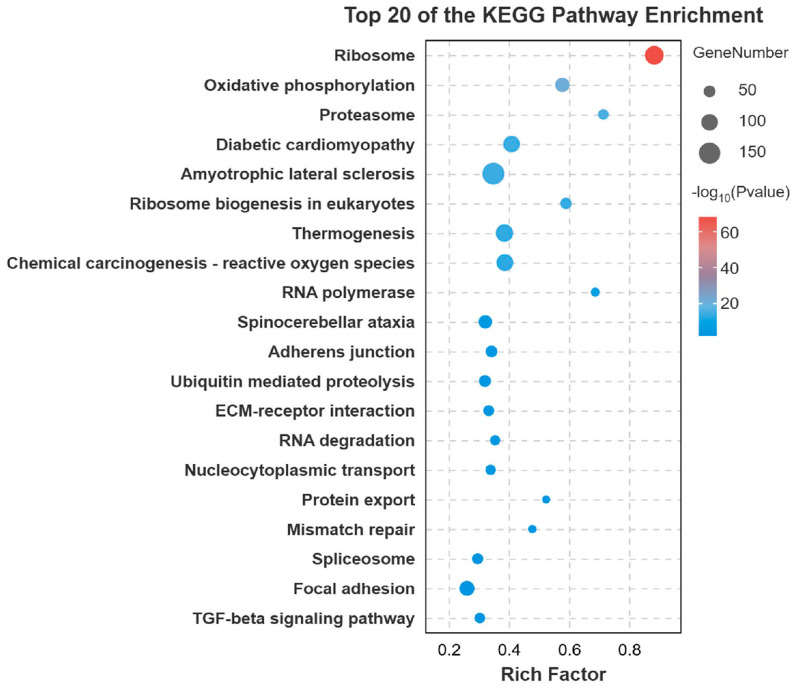
Top 20 KEGG terms for the DEGs. Bubble chart showing the top 20 enriched GO terms. The color scale represents the significance level (e.g., *p*-value), and the bubble size corresponds to the number of enriched genes.

Gene Ontology (GO) enrichment analysis of the DEGs revealed significant enrichment in several key biological processes and molecular functions. The most prominent terms included cellular process, metabolic process, organic substance metabolic process, binding, catalytic activity, and heterocyclic compound binding (Figure 3). To further elucidate the biological pathways involving these DEGs, Kyoto Encyclopedia of Genes and Genomes (KEGG) pathway analysis was performed. The metabolic pathway was the most significantly enriched pathway, encompassing the largest number of DEGs. Other significantly enriched pathways included Endocytosis, Biosynthesis of secondary metabolites, MAPK signaling pathway, Regulation of actin cytoskeleton, and Carbon metabolism (Figure 4).

### 3.6. Identification of Key Candidate Genes for Growth by Integrated Analysis

**Figure 5:**
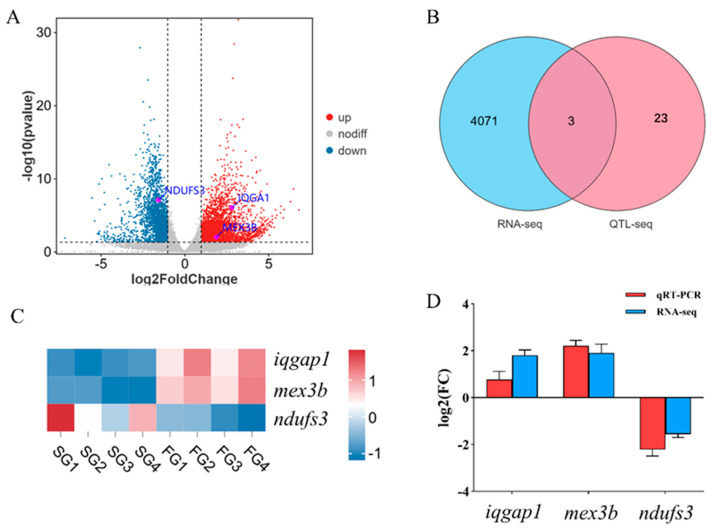
Identification of key genes related to growth. (**A**): Volcano plot of differentially expressed genes (DEGs) in the fast–growing group versus the slow–growing group. (**B**): A Venn diagram showing the intersection of DEGs from the RNA–seq analysis and candidate genes located within significant QTL regions from the QTL–seq analysis. (**C**): Expression profile heatmap of the three candidate genes (*iqgap1*, *mex3b*, *ndufs3*). (**D**): Comparative analysis of gene expression levels obtained from RNA–seq and qRT–PCR.

To identify key candidate genes associated with growth, the results from the QTL-seq and RNA-seq analyses were integrated. This integrated analysis identified three genes—*iqgap1*, *mex3b* and *ndufs3*—that were both located within the major growth-related QTL region on LG02 and were also differentially expressed between the fast-growing and slow-growing group (Figure 5B). The RNA-seq data revealed that the expression levels of *iqgap1* and *mex3b* were significantly up-regulated in the muscle tissue of the FG compared to the SG. In contrast, the expression of *ndufs3* was significantly down-regulated in the fast-growing group (Figure 5A,C). To validate these transcriptomic findings, quantitative real-time PCR (qRT-PCR) was performed. The qRT-PCR results were consistent with the RNA-seq data, confirming the higher expression of *iqgap1* and *mex3b* and the lower expression of *ndufs3* in the fast-growing group (Figure 5D). These converging lines of evidence strongly suggest that *iqgap1*, *mex3b* and *ndufs3* are key genes involved in the regulation of growth in this species.

### 3.7. Validation of Key Candidate Genes in the Rapidly Growing Giant Grouper

**Figure 6:**
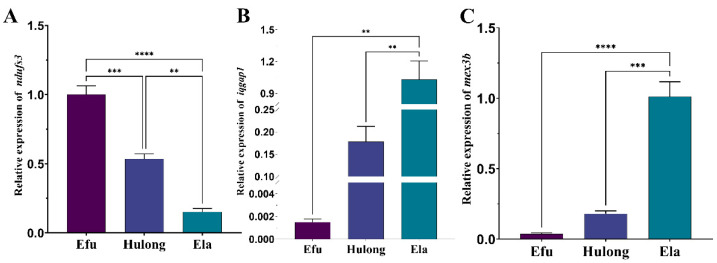
Relative expression of *iqgap1*, *mex3b* and *ndufs3* in the muscle tissue of three grouper types. (**A**) Relative expression of *ndufs3*. (**B**) Relative expression of *iqgap1*. (**C**) Relative expression of *mex3b*.Efu: *Epinephelus fuscoguttatus*; Ela: *Epinephelus lanceolatus*; Hulong: *Epinephelus fuscoguttatus* ♀ × *Epinephelus lanceolatus* ♂. Statistical significance is indicated by asterisks (** *p* < 0.01, *** *p* < 0.001, **** *p* < 0.0001).

To investigate whether the key genes (*iqgap1*, *mex3b*, and *ndufs3*) are associated with the rapid growth of giant grouper, we compared the three genes’ relative expression levels in muscle tissue of hulong hybrid grouper and its parents. The qRT-PCR analysis revealed distinct expression patterns among the three groups (Figure 6). The relative expression levels of *iqgap1* and *mex3b* were significantly higher in the giant grouper compared to both the hulong hybrid and the brown-marbled grouper. Conversely, the expression of *ndufs3* was significantly lower in the giant grouper than in the other two grouper types. These results suggest that *iqgap1*, *mex3b* and *ndufs3* likely play crucial roles in the superior growth performance of the giant grouper.

## 4. Discussion

The hulong hybrid grouper is produced by crossing two parent species with significant differences in growth rates: the giant grouper and the brown-marbled grouper. The self-cross population of hulong hybrid grouper is an ideal material for breeding, as it offers significant advantages for genetic studies, including high genetic diversity, extensive phenotypic segregation, and abundant genetic variation. In this study, we successfully constructed such a self-cross population of hulong hybrid grouper, providing a crucial resource for future grouper breeding. By integrating bulk segregant analysis (BSA) with comparative transcriptomics in this population, our study identified a major quantitative trait locus (QTL) and a set of key candidate genes associated with the growth of giant grouper. More importantly, these findings provide novel insights into the potential genetic mechanisms underlying the superior growth performance of the giant grouper.

### 4.1. Identification of the Growth-Related QTL and SNPs by BSA

Precise localization of genomic regions that control complex traits is one of the purposes of molecular breeding [37,38]. In this study, by leveraging the high recombination rates in the F2 segregating population and the efficiency of the BSA strategy, we successfully narrowed down a major growth-associated QTL to a 2.1 Mb interval on chromosome 2. Unlike previous studies that often reported broad QTL intervals spanning tens of megabases [39,40,41], this refined region containing only 23 annotated genes provides a manageable and focused set of targets for candidate gene discovery. Within this region, the identification of five non-synonymous SNPs is particularly significant. Non-synonymous variants are of distinct biological interest because they directly alter the amino acid sequence, potentially impacting protein structure and function [42,43], which makes them an important cause of the causal mutations underlying the observed growth divergence.

The five identified growth-related SNPs are located in the exons of four genes: adamts10, E.fuscoguttatus.1399, E.fuscoguttatus.14014, E.fuscoguttatus.14170. Among these, adamts10 is a particularly compelling candidate. The ADAMTS family, comprising 19 members, is known to play crucial roles in connective tissue organization and skeletal development [44]. Specifically, adamts6 and adamts10 are involved in fine-tuning fibrillin microfibrils during limb development. These microfibrils, in turn, regulate growth factors of the TGF-β superfamily, including bone morphogenetic proteins (BMPs) and growth/differentiation factors (GDFs). Deficiencies in adamts6 or adamts10 can lead to an abnormal accumulation of fibrillin microfibrils, which impairs downstream BMP signaling and consequently affects normal skeletal formation [45]. The role of adamts10 in skeletal growth and development in both humans and mice strongly supports its potential involvement in regulating fish growth [46,47,48]. The non-synonymous mutation identified in the exon of adamts10 in our study could potentially alter the protein sequence, thereby affecting its function and leading to a slower or hindered skeletal development rate. While the functions of the other three genes containing non-synonymous SNPs remain uncharacterized and warrant further research, the variants identified in this study are of immediate value. These non-synonymous SNPs, strongly associated with growth, represent a valuable resource of molecular markers. They can be integrated into marker-assisted selection (MAS) programs to facilitate the genetic improvement of groupers and breed new strains with superior growth performance.

### 4.2. Differentially Expressed Genes Associated with Extreme Growth Rates

Skeletal muscle constitutes 40–50% of the total body weight in fish, and its mass accretion is a direct determinant of individual growth. This process is intricately regulated by a variety of factors, including growth hormone (GH), insulin-like growth factors (IGFs), and myogenic regulatory factors like MYF5, MYOD, and MRF4 [48]. By comparing the muscle transcriptomes of the FG and SG groups from the self-cross population of the hulong hybrid grouper, our study identified numerous differentially expressed genes (DEGs) that likely contribute to their divergent growth phenotypes.

A key finding was the significant up-regulation of genes within the GH/IGF axis in the FG group, most notably the growth hormone receptor (*ghr*) and insulin-like growth factor-binding protein 3 (*igfbp3*). The GH/IGF axis is the principal endocrine regulator of somatic growth. GHR initiates intracellular signaling upon binding GH, which stimulates the synthesis and secretion of IGFs from the liver. These IGFs then circulate to target tissues, promoting cell growth and differentiation, and ultimately driving organ and body growth [49]. Concurrently, IGFBPs, such as IGFBP3, modulate IGF activity by binding to IGFs, thereby extending their half-life and creating a stable, circulating reservoir [50]. Upon reaching target tissues, IGFBPs are proteolytically cleaved, releasing IGFs from the complex. This increases the availability of free IGFs for binding to their receptor (IGF1R), thus amplifying the pro-growth IGF signal [51]. The importance of this family is underscored by studies showing that mice with a knockout of the igfbp4 gene are significantly smaller than their wild-type counterparts [52]. The elevated expression of ghr and igfbp3 in the FG group suggests a more robust and efficient GH/IGF signaling cascade, which likely underpins their accelerated growth.

Conversely, we observed a significant down-regulation of the cytokine-inducible SH2-containing protein (CISH) gene in the FG group. CISH is known to act as a negative regulator of cytokine receptor signaling by binding to tyrosine-phosphorylated receptors via its central SH2 domain. Previous research has demonstrated that overexpression of *cish* can reduce the proliferation rate of fibroblasts [53]. Therefore, the lower expression of *cish* in the fast-growing grouper likely results in weaker negative feedback on growth-related cytokine signaling pathways, contributing to a more sustained proliferative state and consequently, a faster growth rate.

Furthermore, DEG analysis identified several other genes previously reported to be involved in growth and development, including *igf1r* [54], *fgfr4* [55], *ccn1* [56], and *crim1* [57]. The differential expression of this suite of genes highlights the complexity of growth regulation and indicates that growth is controlled by the coordinated action of multiple genes.

### 4.3. Key Genes Potentially Driving Rapid Growth in the Giant Grouper

Through an integrated analysis of QTL-seq and RNA-seq data, this study identified three key genes—*iqgap1*, *mex3b* and *ndufs3*—strongly associated with the rapid growth of the giant grouper. The distinct expression patterns and known functions of these genes provide valuable insights into the molecular mechanisms underlying this growth trait.

*iqgap1* encodes a scaffold protein known to regulate multiple signaling pathways critical for growth and development by directly interacting with various target proteins. Its functions span cytoskeleton organization, cell migration and proliferation, actin dynamics, and neurite outgrowth promotion [58,59,60]. Studies have shown that IQGAP1 can bind to Raf and MEK, thereby enhancing ERK signaling and promoting cell proliferation [61]. In our study, the significantly higher relative expression of *iqgap1* in the giant grouper, compared to the brown-marbled and hulong hybrid groupers, strongly suggests that it plays a pivotal role in driving its superior growth, likely through the potentiation of pro-proliferative signaling pathways like the ERK/MAPK cascade.

The *ndufs3* gene encodes a core subunit of mitochondrial Complex I, a critical enzyme in the mitochondrial respiratory chain responsible for cellular energy metabolism. The NDUFS3 protein is essential for the proper assembly of Complex I and for the electron transport chain’s efficiency in generating ATP [62]. As ATP is the direct energy currency for nearly all life activities, including cell proliferation and growth, an adequate supply is paramount. Interestingly, our results showed that *ndufs3* expression was significantly lower in the fast-growing giant grouper. Studies have shown that the “Warburg effect” is observed in rapidly proliferating cells, which is characterized by a decrease in mitochondrial oxidative phosphorylation and an enhancement of glycolysis [63]. This process can generate more biosynthetic precursors, such as 3-phosphoglycerate and pyruvate [64]. Therefore, the reduced expression level of *ndufs3* in the Giant grouper might promote glycolysis to ensure the supply of raw materials for rapid cell growth, thereby promoting individual growth.

MEX3B is an RNA-binding protein that post-transcriptionally regulates gene expression by binding to the 3’ UTR of specific mRNAs. Its known functions are diverse, including inducing apoptosis by up-regulating the pro-apoptotic protein BIM [65] and acting as a co-receptor to facilitate TLR3-mediated innate immune responses. The MEX3 protein family was first identified in Caenorhabditis elegans [66], where it plays a critical role in embryonic and post-embryonic development [67]. Although our study identified *mex3b* as a growth-related gene through its differential expression, its direct role in regulating fish growth has not yet been reported. The mechanisms by which *mex3b* influences growth remain to be elucidated and warrant further investigation.

## 5. Conclusions

In this study, we successfully constructed a self-cross population of the hulong hybrid grouper, and utilized this population to investigate the genetic basis of growth in giant grouper. The results showed that a major candidate QTL on chromosome 2 was significantly associated with growth, and seven SNPs were associated with growth within the candidate QTL. Then we combined BSA and RNA-seq results to identify three critical genes (*iqgap1*, *mex3b* and *ndufs3*) associated with the growth of the giant grouper. Subsequent validation by qRT-PCR confirmed that the expression levels of these three genes were significantly different in giant grouper compared to the brown-marbled and hulong hybrid groupers, strongly suggesting their involvement in the superior growth performance of giant grouper. Collectively, our findings provide novel insights into the molecular mechanisms governing growth regulation and identify valuable genetic markers for grouper breeding.

## Figures and Tables

**Table 1:** Statistics of growth traits of the self-cross population of hulong hybrid grouper.

	W/g	BL/mm	TL/mm	BH/mm	BW/mm
Mean	7.72	63.93	73.92	21.07	10.51
SE	0.11	0.32	0.35	0.11	0.07
SD	3.73	10.98	12.18	3.87	2.41
Min	1.3	36.9	23.86	10.43	3.53
Max	28.16	99.32	113.69	33.93	19.52
CV%	48.32	17.18	16.48	18.37	22.93

SE: standard error; SD: standard deviation; CV: coefficient of variation.

**Table 2:** Phenotypic correlation for the five growth traits.

	BL	TL	BH	BW	W
BL	1.00				
TL	0.93 *	1.00			
BH	0.92 *	0.96 *	1.00		
BW	0.88 *	0.89 *	0.88 *	1.00	
W	0.80 *	0.81 *	0.80 *	0.82 *	1.00

* *p* < 0.05.

**Table 3:** Growth traits of the fast-growing and slow-growing groups.

	W/g	BL/mm	TL/mm	BH/mm	BW/mm
Fast-growing (FG)	16.14 ± 2.56	84.87 ± 3.92	97.85 ± 4.53	27.31 ± 2.41	14.50 ± 1.47
Slow-growing (SG)	2.56 ± 1.30	45.05 ± 5.87	52.29 ± 4.88	14.90 ± 2.22	6.93 ± 1.28

**Table 4:** Annotation of genes within the significant region.

Gene_ID	Chr	Star (bp)	End (bp)	Gene
E.fuscoguttatus.13898	LG02	1,073,355	1,077,638	*cnih3*
E.fuscoguttatus.13901	LG02	1,115,269	1,188,685	*tmc3*
E.fuscoguttatus.13920	LG02	1,281,285	1,282,184	*cartpt*
E.fuscoguttatus.13924	LG02	1,352,922	1,354,123	\
E.fuscoguttatus.13932	LG02	1,442,340	1,443,422	*gorab*
E.fuscoguttatus.13934	LG02	1,452,097	1,526,678	*iqgap1*
E.fuscoguttatus.13973	LG02	1,541,299	1,685,587	*crtc3*
E.fuscoguttatus.13989	LG02	1,794,496	1,797,849	*mex3b*
E.fuscoguttatus.13992	LG02	1,832,256	1,847,559	\
E.fuscoguttatus.14014	LG02	1,915,601	1,926,667	\
E.fuscoguttatus.14020	LG02	2,102,640	2,109,601	*c1qtnf4*
E.fuscoguttatus.14027	LG02	2,139,273	2,187,493	*ndufs3*
E.fuscoguttatus.14035	LG02	2,195,104	2,211,641	*ptpmt1*
E.fuscoguttatus.14040	LG02	2,222,780	2,255,397	*prc1*
E.fuscoguttatus.14054	LG02	2,256,550	2,296,184	*vps-33.2*
E.fuscoguttatus.14078	LG02	2,296,711	2,307,568	*hddc3*
E.fuscoguttatus.14083	LG02	2,329,027	2,339,191	*mfap1*
E.fuscoguttatus.14092	LG02	2,380,592	2,385,958	\
E.fuscoguttatus.14096	LG02	2,485,216	2,509,566	*serinc4*
E.fuscoguttatus.14104	LG02	2,515,814	2,600,891	*adamtsl5*
E.fuscoguttatus.14114	LG02	2,650,301	2,699,284	*adamts10*
E.fuscoguttatus.14121	LG02	2,828,330	2,829,502	*thap5*
E.fuscoguttatus.14124	LG02	3,029,596	3,141,749	*efl1*

**Table 5:** The validation of SNPs related to growth.

SNP	Allele	Allele Frequency		*p* Values (Allele Frequency)	Genotype	Genotype Frequency		*p* Values (Genotype Frequency
		Fast-Growing	Slow-Growing			Fast-Growing	Slow-Growing	
LG02__1842286	C	76	48	0.0138	CC	35	18	0.0377
	A	8	16		CA	6	12	
					AA	1	2	
LG02__1844809	G	73	49	0.0071	GG	33	20	0.0329
	A	7	17		GA	7	9	
					AA	0	4	
LG02__1919581	T	71	51	0.0348	TT	32	23	0.0418
	C	7	15		TC	7	5	
					CC	0	5	
LG02__1926647	T	78	56	0.0102	TT	36	24	0.0437
	C	6	16		TC	6	8	
					CC	0	4	
LG02__2665634	C	75	54	0.0128	CC	35	20	0.0049
	T	5	14		CT	5	14	

## Data Availability

The raw sequence reads have been deposited in Genome Sequence Archive (GSA)’s BioProject Accession PRJCA046310.

## Supplementary Materials

The following supporting information can be downloaded at: https://www.mdpi.com/article/10.3390/ani15243599/s1, Table S1: qRT-PCR primer sequences; Table S2: Summary of whole-genome resequencing data for the two extreme bulks; Table S3: Allele and genotype frequencies of non-synonymous SNPs within the candidate QTL region in the fast-growing and slow-growing bulks; Table S4: Quality statistics of RNA-seq data for the eight muscle tissue samples; Figure S1: Representative appearance of self-cross offspring of hulong hybrid grouper (E. fuscoguttatus ♀ × E. lanceolatus ♂); Figure S2: Quantile-Quantile (QQ) plots for assessing the normality of distribution for five growth-related traits; Figure S3: Distribution of SNPs in chromosomes of self-cross population of hulong hybrid grouper; Figure S4: Δ(SNP-index) plot of the candidate region on LG02; Figure S5: G′ value profile on LG02.

## References

[1] Zhang Y., Zhuo H., Fu S., Liu J. (2024). Growth performance and growth model fitting of Litopenaeus vannamei cultured in pond and factory modes. Aquac. Rep..

[2] Yoshida G.M., Yáñez J.M. (2021). Multi-trait GWAS using imputed high-density genotypes from whole-genome sequencing identifies genes associated with body traits in Nile tilapia. BMC Genom..

[3] Ranke M.B., Wit J.M. (2018). Growth hormone-past, present and future. Nat. Rev. Endocrinol..

[4] Alzaid A., Kim J., Devlin R., Martin S., Macqueen D. (2018). Growth hormone transgenesis in coho salmon disrupts muscle immune function impacting cross-talk with growth systems. J. Exp. Biol..

[5] Canosa L., Bertucci J. (2020). Nutrient regulation of somatic growth in teleost fish. The interaction between somatic growth, feeding and metabolism. Mol. Cell. Endocrinol..

[6] Li N., Zhou T., Geng X., Jin Y., Wang X., Liu S., Xu X., Gao D., Li Q., Liu Z. (2018). Identification of novel genes significantly affecting growth in catfish through GWAS analysis. Mol. Genet. Genom..

[7] Zhang D., Luo L., Wang Z., Yu Y., Nie C., Guo X., Gao Z. (2024). Identification of novel SNPs and candidate genes significantly affecting growth in grass carp (*Ctenopharyngodon idella*) through GWAS analysis. Aquaculture.

[8] Horn S.S., Ruyter B., Meuwissen T.H.E., Moghadam H., Hillestad B., Sonesson A.K. (2020). GWAS identifies genetic variants associated with omega-3 fatty acid composition of Atlantic salmon fillets. Aquaculture.

[9] Verrier E.R., Genet C., Laloë D., Jaffrezic F., Rau A., Esquerre D., Dechamp N., Ciobotaru C., Hervet C., Krieg F. (2018). Genetic and transcriptomic analyses provide new insights on the early antiviral response to VHSV in resistant and susceptible rainbow trout. BMC Genom..

[10] Chen G., Liu H., Yu X., Luo W., Tong J. (2023). Estimation of heritabilities and quantitative trait loci for growth traits of bighead carp (*Hypophthalmichthys nobilis*). Aquaculture.

[11] Mackay T.F.C. (2014). Epistasis and quantitative traits: Using model organisms to study gene–gene interactions. Nat. Rev. Genet..

[12] Phillips P.C. (2008). Epistasis—The essential role of gene interactions in the structure and evolution of genetic systems. Nat. Rev. Genet..

[13] Keurentjes J.J.B., Willems G., van Eeuwijk F., Nordborg M., Koornneef M. (2011). A comparison of population types used for QTL mapping in Arabidopsis thaliana. Plant Genet. Resour..

[14] Wang S., Wang Y., Li Y., Xiao F., Guo H., Gao H., Wang N., Zhang H., Li H. (2022). Genome-Wide Association Study and Selective Sweep Analysis Reveal the Genetic Architecture of Body Weights in a Chicken F(2) Resource Population. Front. Vet. Sci..

[15] Collard B.C.Y., Jahufer M.Z.Z., Brouwer J.B., Pang E.C.K. (2005). An introduction to markers, quantitative trait loci (QTL) mapping and marker-assisted selection for crop improvement: The basic concepts. Euphytica.

[16] Casiró S., Velez-Irizarry D., Ernst C.W., Raney N.E., Bates R.O., Charles M.G., Steibel J.P. (2017). Genome-wide association study in an F2 Duroc x Pietrain resource population for economically important meat quality and carcass traits. J. Anim. Sci..

[17] Zhou Z., Li M., Cheng H., Fan W., Yuan Z., Gao Q., Xu Y., Guo Z., Zhang Y., Hu J. (2018). An intercross population study reveals genes associated with body size and plumage color in ducks. Nat. Commun..

[18] Feng X., Yu X., Fu B., Wang X., Liu H., Pang M., Tong J. (2018). A high-resolution genetic linkage map and QTL fine mapping for growth-related traits and sex in the Yangtze River common carp (*Cyprinus carpio haematopterus*). BMC Genom..

[19] Yang Y., Wu L., Wu X., Li B., Huang W., Weng Z., Lin Z., Song L., Guo Y., Meng Z. (2020). Identification of Candidate Growth-Related SNPs and Genes Using GWAS in Brown-Marbled Grouper (*Epinephelus fuscoguttatus*). Mar. Biotechnol..

[20] Williams K.C. (2009). A review of feeding practices and nutritional requirements of postlarval groupers. Aquaculture.

[21] Chen Z.-F., Tian Y.-S., Wang P.-F., Tang J., Liu J.-C., Ma W.-H., Li W.-S., Wang X.-M., Zhai J.-M. (2018). Embryonic and larval development of a hybrid between kelp grouper *Epinephelus moara* ♀ × giant grouper *E. lanceolatus* ♂ using cryopreserved sperm. Aquac. Res..

[22] Nankervis L., Cobcroft J.M., Nguyen N.V., Rimmer M.A. (2022). Advances in practical feed formulation and adoption for hybrid grouper (*Epinephelus fuscoguttatus* ♀ × *E. lanceolatus* ♂) aquaculture. Rev. Aquac..

[23] Gong S., Wang T., Cai C., Cai J., Yang Y., Zhong C., Wu X., Tao Y., Zeng L., Wei Q. (2025). Development and characterization of a novel intergeneric hybrid grouper (*Cromileptes altivelis* ♀ × *Epinephelus lanceolatus* ♂). Aquaculture.

[24] Wu L., Yang Y., Li B., Huang W., Wang X., Liu X., Meng Z., Xia J. (2019). First Genome-wide Association Analysis for Growth Traits in the Largest Coral Reef-Dwelling Bony Fishes, the Giant Grouper (*Epinephelus lanceolatus*). Mar. Biotechnol..

[25] Wu L., Yang Y., Wang X., Weng Z., Hua S., Li D., Xia J., Liu X., Meng Z. (2023). Genome-wide QTL mapping and RNA-seq reveal the genetic variation influencing growth traits in giant grouper (*Epinephelus lanceolatus*). Aquaculture.

[26] Chen S. (2023). Ultrafast one-pass FASTQ data preprocessing, quality control, and deduplication using fastp. Imeta.

[27] Jung Y., Han D. (2022). BWA-MEME: BWA-MEM emulated with a machine learning approach. Bioinformatics.

[28] Danecek P., Bonfield J.K., Liddle J., Marshall J., Ohan V., Pollard M.O., Whitwham A., Keane T., McCarthy S.A., Davies R.M. (2021). Twelve years of SAMtools and BCFtools. Gigascience.

[29] Van der Auwera G.A., Carneiro M.O., Hartl C., Poplin R., Del Angel G., Levy-Moonshine A., Jordan T., Shakir K., Roazen D., Thibault J. (2013). From FastQ data to high confidence variant calls: The Genome Analysis Toolkit best practices pipeline. Curr. Protoc. Bioinform..

[30] Danecek P., Auton A., Abecasis G., Albers C.A., Banks E., DePristo M.A., Handsaker R.E., Lunter G., Marth G.T., Sherry S.T. (2011). The variant call format and VCFtools. Bioinformatics.

[31] Takagi H., Abe A., Yoshida K., Kosugi S., Natsume S., Mitsuoka C., Uemura A., Utsushi H., Tamiru M., Takuno S. (2013). QTL-seq: Rapid mapping of quantitative trait loci in rice by whole genome resequencing of DNA from two bulked populations. Plant J..

[32] Mansfeld B.N., Grumet R. (2018). QTLseqr: An R Package for Bulk Segregant Analysis with Next-Generation Sequencing. Plant Genome.

[33] Zhang Y., Park C., Bennett C., Thornton M., Kim D. (2021). Rapid and accurate alignment of nucleotide conversion sequencing reads with HISAT-3N. Genome Res..

[34] Liao Y., Smyth G.K., Shi W. (2014). featureCounts: An efficient general purpose program for assigning sequence reads to genomic features. Bioinformatics.

[35] Love M.I., Huber W., Anders S. (2014). Moderated estimation of fold change and dispersion for RNA-seq data with DESeq2. Genome Biol..

[36] Rao X., Huang X., Zhou Z., Lin X. (2013). An improvement of the 2ˆ(-delta delta CT) method for quantitative real-time polymerase chain reaction data analysis. Biostat Bioinforma Biomath.

[37] Doerge R.W. (2002). Mapping and analysis of quantitative trait loci in experimental populations. Nat. Rev. Genet..

[38] Yue G.H. (2014). Recent advances of genome mapping and marker-assisted selection in aquaculture. Fish Fish..

[39] Zhu C., Liu H., Pan Z., Chang G., Wang H., Wu N., Ding H., Yu X. (2019). Construction of a high-density genetic linkage map and QTL mapping for growth traits in Pseudobagrus ussuriensis. Aquaculture.

[40] Li C., Wang J., Song K., Meng J., Xu F., Li L., Zhang G. (2018). Construction of a high-density genetic map and fine QTL mapping for growth and nutritional traits of Crassostrea gigas. BMC Genom..

[41] Huang X., Jiang Y., Zhang W., Cheng Y., Wang Y., Ma X., Duan Y., Xia L., Chen Y., Wu N. (2020). Construction of a high-density genetic map and mapping of growth related QTLs in the grass carp (Ctenopharyngodon idellus). BMC Genom..

[42] Tokuriki N., Tawfik D.S. (2009). Stability effects of mutations and protein evolvability. Curr. Opin. Struct. Biol..

[43] Martincorena I., Raine K.M., Gerstung M., Dawson K.J., Haase K., Van Loo P., Davies H., Stratton M.R., Campbell P.J. (2017). Universal Patterns of Selection in Cancer and Somatic Tissues. Cell.

[44] Apte S.S. (2009). A disintegrin-like and metalloprotease (reprolysin-type) with thrombospondin type 1 motif (ADAMTS) superfamily: Functions and mechanisms. J. Biol. Chem..

[45] Mead T.J., Martin D.R., Wang L.W., Cain S.A., Gulec C., Cahill E., Mauch J., Reinhardt D., Lo C., Baldock C. (2022). Proteolysis of fibrillin-2 microfibrils is essential for normal skeletal development. Elife.

[46] Dagoneau N., Benoist-Lasselin C., Huber C., Faivre L., Mégarbané A., Alswaid A., Dollfus H., Alembik Y., Munnich A., Legeai-Mallet L. (2004). ADAMTS10 mutations in autosomal recessive Weill-Marchesani syndrome. Am. J. Hum. Genet..

[47] Taye N., Karoulias S.Z., Balic Z., Wang L.W., Willard B.B., Martin D., Richard D., Okamoto A.S., Capellini T.D., Apte S.S. (2025). Combined ADAMTS10 and ADAMTS17 inactivation exacerbates bone shortening and compromises extracellular matrix formation. bioRxiv.

[48] Bentzinger C.F., Wang Y.X., Rudnicki M.A. (2012). Building muscle: Molecular regulation of myogenesis. Cold Spring Harb. Perspect. Biol..

[49] Yang S., Liu Z., Yan Z., Zhao Z., Zhang C., Gong Q., Du X., Wu J., Feng Y., Du J. (2021). Improvement of skeletal muscle growth by GH/IGF growth-axis contributes to growth performance in commercial fleshy sturgeon. Aquaculture.

[50] Rajaram S., Baylink D.J., Mohan S. (1997). Insulin-like growth factor-binding proteins in serum and other biological fluids: Regulation and functions. Endocr. Rev..

[51] Allard J.B., Duan C. (2018). IGF-Binding Proteins: Why Do They Exist and Why Are There So Many?. Front. Endocrinol..

[52] Ning Y., Schuller A.G., Conover C.A., Pintar J.E. (2008). Insulin-like growth factor (IGF) binding protein-4 is both a positive and negative regulator of IGF activity in vivo. Mol. Endocrinol..

[53] Uchida K., Yoshimura A., Inazawa J., Yanagisawa K., Osada H., Masuda A., Saito T., Takahashi T., Miyajima A., Takahashi T. (1997). Molecular cloning of CISH, chromosome assignment to 3p21.3, and analysis of expression in fetal and adult tissues. Cytogenet. Cell Genet..

[54] Chitnis M.M., Yuen J.S., Protheroe A.S., Pollak M., Macaulay V.M. (2008). The type 1 insulin-like growth factor receptor pathway. Clin. Cancer Res..

[55] Citores L., Bai L., Sørensen V., Olsnes S. (2007). Fibroblast growth factor receptor-induced phosphorylation of STAT1 at the Golgi apparatus without translocation to the nucleus. J. Cell. Physiol..

[56] Su J.L., Chiou J., Tang C.H., Zhao M., Tsai C.H., Chen P.S., Chang Y.W., Chien M.H., Peng C.Y., Hsiao M. (2010). CYR61 regulates BMP-2-dependent osteoblast differentiation through the {alpha}v{beta}3 integrin/integrin-linked kinase/ERK pathway. J. Biol. Chem..

[57] Wilkinson L., Kolle G., Wen D., Piper M., Scott J., Little M. (2003). CRIM1 regulates the rate of processing and delivery of bone morphogenetic proteins to the cell surface. J. Biol. Chem..

[58] Li Z., McNulty D.E., Marler K.J., Lim L., Hall C., Annan R.S., Sacks D.B. (2005). IQGAP1 promotes neurite outgrowth in a phosphorylation-dependent manner. J. Biol. Chem..

[59] Briggs M.W., Sacks D.B. (2003). IQGAP proteins are integral components of cytoskeletal regulation. EMBO Rep..

[60] Brown M.D., Sacks D.B. (2006). IQGAP1 in cellular signaling: Bridging the GAP. Trends Cell Biol..

[61] Ren J.G., Li Z., Sacks D.B. (2007). IQGAP1 modulates activation of B-Raf. Proc. Natl. Acad. Sci. USA.

[62] Bénit P., Slama A., Cartault F., Giurgea I., Chretien D., Lebon S., Marsac C., Munnich A., Rötig A., Rustin P. (2004). Mutant NDUFS3 subunit of mitochondrial complex I causes Leigh syndrome. J. Med. Genet..

[63] Vander Heiden M.G., Cantley L.C., Thompson C.B. (2009). Understanding the Warburg effect: The metabolic requirements of cell proliferation. Science.

[64] Kroemer G., Pouyssegur J. (2008). Tumor cell metabolism: Cancer’s Achilles’ heel. Cancer Cell.

[65] Oda T., Yamazumi Y., Hiroko T., Kamiya A., Kiriya S., Suyama S., Shiozaki-Sato Y., Akiyama T. (2018). Mex-3B induces apoptosis by inhibiting miR-92a access to the Bim-3’UTR. Oncogene.

[66] Yang Y., Wang S.Y., Huang Z.F., Zou H.M., Yan B.R., Luo W.W., Wang Y.Y. (2016). The RNA-binding protein Mex3B is a coreceptor of Toll-like receptor 3 in innate antiviral response. Cell Res..

[67] Pagano J.M., Farley B.M., Essien K.I., Ryder S.P. (2009). RNA recognition by the embryonic cell fate determinant and germline totipotency factor MEX-3. Proc. Natl. Acad. Sci. USA.

