# Supplementary material for: Identification of Genes Related to Rapid Growth of Giant Grouper (Epinephelus lanceolatus) Based on Self-Cross Population of Hulong Hybrid Grouper (E. fuscoguttatus ♀ × E. lanceolatus ♂)
Source: Animals (Basel). 2025 Dec 15;15(24):3599. doi: 10.3390/ani15243599 (PMC12729531; doi:10.3390/ani15243599)
Supplement: Supplementary file 1 [file animals-15-03599-s001.zip › animals-4023678-supplementary.pdf]

Table S1 qRT-PCR primer sequences

Gene	Primer Sequences (5'->3')	Tm(°C)	Efficiency (%)	Product Length
iqgap1	F: AGAGAAGATGAAGCAGGTGAA	56.65	95.6	180
	R: CGATACCGTCTCTGATTCCTTATG	58.61		
mex3b	F: GAGCAAAGACCAACACCTACA	58.15	98.3	173
	R: GCTCCCATTCAGACCAGTATTT	58.11		
ndufs3	F: AGAACCGCTTTGAGATTGTGTA	58.00	96.7	183
	R: GGTCTGGGTGATTTGAGAAGAA	57.98		
β-actin	F: GATCTGGCATCACACCTTCTAC	58.54	99.4	218
	R: CACCGGAGTCCATGACAATAC	58.45		

Table S2 Summary of whole-genome resequencing data

Sample	Clean reads	GC (%)	Q30 (%)	Mapping rate (%)	Mean depth
SG_1	100,561,526	95.96	41.35	\	\
SG_2	95,131,758	96.96	41.35	\	\
SG_3	110,912,228	95.82	41.34	\	\
SG_4	89,823,598	96.13	41.29	\	\
SG_5	94,021,362	95.29	41.42	\	\
SG_6	107,966,348	95.77	41.35	\	\
SG_7	102,055,394	95.36	41.44	\	\
SG_8	107,426,286	96.25	41.36	\	\
SG_9	111,456,516	96.18	41.27	\	\
SG_10	108,561,078	96.15	41.31	\	\
SG_11	121,267,682	95.99	41.38	\	\
SG_12	113,202,948	96.16	41.31	\	\
SG_13	112,304,474	96.05	41.36	\	\
SG_14	77,625,382	95.88	41.27	\	\
SG_15	89,203,986	95.76	41.30	\	\
SG_16	114,729,736	95.27	41.36	\	\
SG_17	112,135,148	96.29	41.43	\	\
SG_18	104,973,246	96.70	41.24	\	\

SG_19	63,605,722	95.85	41.52	\	\
SG_20	103,490,570	96.05	41.33	\	\
SG_21	106,662,542	96.30	41.26	\	\
SG_22	112,629,538	96.13	41.39	\	\
SG_23	94,659,784	95.47	41.39	\	\
SG_24	110,879,772	95.83	41.31	\	\
SG_25	111,957,634	95.98	41.26	\	\
SG_26	91,504,766	96.13	41.39	\	\
SG_27	107,194,818	96.36	41.34	\	\
SG_28	96,606,106	96.36	41.34	\	\
SG_29	86,540,170	96.21	41.28	\	\
SG_30	80,511,596	96.13	41.26	\	\
SG_31	79,099,270	96.19	41.27	\	\
SG_32	117,802,652	96.11	41.36	\	\
SG_33	125,406,274	95.88	41.35	\	\
SG_34	103,031,368	96.23	41.33	\	\
SG_35	97,815,310	95.99	41.35	\	\
SG_36	113,804,958	96.67	41.28	\	\
SG_37	117,191,342	95.65	41.60	\	\
SG_38	129,109,472	96.02	41.29	\	\
SG_39	122,403,540	95.95	41.33	\	\
SG_40	116,058,506	96.12	41.42	\	\
SG_41	95,559,098	95.70	41.36	\	\
SG_42	103,722,976	95.81	41.29	\	\
SG_43	103,387,500	96.29	41.31	\	\
SG_44	78,666,232	96.07	41.42	\	\
SG_45	92,263,972	95.17	41.47	\	\
SG_46	118,570,830	96.64	41.37	\	\
SG_47	110,601,908	96.41	41.33	\	\

SG_48	96,272,978	96.48	41.23	\	\
SG_49	100,093,016	96.04	41.26	\	\
SG_50	107,890,182	96.47	41.26	\	\
SG_51	111,876,512	96.38	41.36	\	\
SG_52	104,324,836	96.48	41.26	\	\
SG_53	122,065,222	96.41	41.29	\	\
SG_54	124,508,156	96.39	41.51	\	\
SG_55	91,047,134	96.17	41.25	\	\
SG_56	118,407,696	95.75	41.57	\	\
SG_57	106,831,780	96.27	41.31	\	\
SG_58	126,455,258	96.51	41.39	\	\
SG_59	103,762,426	95.91	41.37	\	\
SG_60	113,704,210	96.27	41.37	\	\
FG_1	99,442,082	96.15	41.32	\	\
FG_2	113,966,150	96.04	41.32	\	\
FG_3	95,485,338	96.13	41.33	\	\
FG_4	111,277,018	96.13	41.33	\	\
FG_5	96,997,138	95.89	41.31	\	\
FG_6	106,039,046	96.02	41.33	\	\
FG_7	106,567,334	96.03	41.40	\	\
FG_8	101,874,278	93.78	41.41	\	\
FG_9	69,836,164	96.19	41.56	\	\
FG_10	109,148,462	96.31	41.25	\	\
FG_11	123,204,350	95.80	41.38	\	\
FG_12	84,577,798	93.85	41.00	\	\
FG_13	85,165,984	95.52	41.59	\	\
FG_14	107,814,500	96.54	41.29	\	\
FG_15	116,212,562	96.07	41.36	\	\
FG_16	109,059,616	95.98	41.43	\	\

FG_17	108,696,546	96.30	41.39	\	\
FG_18	124,321,666	96.37	41.28	\	\
FG_19	89,226,922	95.55	41.41	\	\
FG_20	93,722,970	95.89	41.32	\	\
FG_21	111,350,118	96.04	41.28	\	\
FG_22	90,471,186	96.60	41.26	\	\
FG_23	106,478,128	96.31	41.33	\	\
FG_24	120,497,404	95.59	41.63	\	\
FG_25	108,231,234	95.72	41.37	\	\
FG_26	77,507,118	95.24	41.44	\	\
FG_27	124,569,542	96.26	41.34	\	\
FG_28	181,821,038	95.70	41.51	\	\
FG_29	124,015,846	96.62	41.30	\	\
FG_30	94,379,046	95.69	41.54	\	\
FG_31	86,723,662	96.43	41.28	\	\
FG_32	148,473,678	96.22	41.48	\	\
FG_33	91,201,072	95.26	41.31	\	\
FG_34	159,086,348	96.02	41.67	\	\
FG_35	99,470,424	96.01	41.32	\	\
FG_36	127,094,174	95.61	41.61	\	\
FG_37	99,493,276	96.36	41.33	\	\
FG_38	120,088,796	93.91	41.08	\	\
FG_39	117,455,572	95.40	41.50	\	\
FG_40	125,072,286	96.00	41.37	\	\
FG_41	106,758,260	96.08	41.81	\	\
FG_42	91,612,624	95.26	41.32	\	\
FG_43	119,782,498	93.86	41.23	\	\
FG_44	88,523,680	94.22	41.02	\	\
FG_45	151,729,394	95.73	41.50	\	\

FG_46	180,213,644	95.90	41.50	\	\
FG_47	160,840,520	95.36	41.50	\	\
FG_48	94,709,216	95.91	41.48	\	\
FG_49	114,801,968	96.23	41.50	\	\
FG_50	101,080,126	95.79	41.36	\	\
FG_51	82,843,284	96.15	41.32	\	\
FG_52	109,992,792	96.02	41.29	\	\
FG_53	94,634,260	95.49	41.49	\	\
FG_54	145,795,912	95.86	41.68	\	\
FG_55	117,140,302	95.95	41.29	\	\
FG_56	111,110,148	96.04	41.31	\	\
FG_57	128,506,196	96.38	41.26	\	\
FG_58	130,803,338	96.38	41.32	\	\
FG_59	114,059,056	95.76	41.38	\	\
FG_60	112,350,674	96.12	41.35	\	\
Fast-growing-pool	6,291,322,954	41.39	95.82	99.44	813.55
Slow-growing-pool	6,723,905,810	41.34	96.10	99.43	873.71
F	94,314,346	41.78	92.55	99.75	13.37

Note: F represents the maternal parent.

Table S3 Allele and genotype frequencies of non-synonymous SNPs within the candidate QTL region in the fast-growing and slow-growing bulks.

Gene	SNP	Allele	Allele frequenc		P values (Allele frequency)	Genotype	Genotype frequency		P values (Genotype frequency)
			Fast-growing	Slow-growing			Fast-growing	Slow-growing	
cnih3	LG02_1073510	G	41	54	0.0862	TT	23	14	0.1441
		T	79	66		TG	33	38	
						GG	4	8	
tmc3	LG02_1174043	G	114	108	0.1414	GG	54	49	0.3258
		T	6	12		GT	6	10	
						TT	0	1	
	LG02_1174071	A	117	117	1	AA	57	57	1

		G	3	3		AG	3	3	
cartpt	LG02_1282001	C	70	64	0.4354	CC	19	11	0.1545
		T	50	56		CT	32	42	
						TT	9	7	
	LG02_1282139	C	43	55	0.1150	CC	22	14	0.2071
		T	77	65		CT	33	37	
						TT	5	9	
	LG02_1361130	T	116	117	0.7013	TT	56	57	0.6969
		C	4	3		TC	4	3	
gorab	LG02_1442827	A	99	101	0.9754	AA	49	50	0.9997
		G	5	5		AG	1	1	
						GG	2	2	
nrip1	LG02_1474878	A	68	66	0.7949	AA	17	13	0.5300
		C	52	54		AC	34	40	
						CC	9	7	
crtc3	LG02_1623510	G	67	65	0.7952	GG	16	12	0.5233
		T	53	55		GT	35	41	
						TT	9	7	
\	LG02__1841836	C	66	74	0.2949	CC	8	16	0.1862
		T	54	46		CT	50	42	
						TT	2	2	
	LG02_1842286	C	98	112	0.0063	CC	38	52	0.0032
		A	22	8		CA	22	8	
	LG02_1844809	G	38	52	0.0032	GG	98	112	0.0063
		A	22	8		GA	22	8	
	LG02_1845194	A	59	68	0.2445	AA	8	15	0.2607
		G	61	52		AG	43	38	
						GG	9	7	
	LG02_1845559	G	58	68	0.1961	GG	7	15	0.1655
		T	62	52		GT	44	38	
						GG	9	7	
	LG02_1846079	C	98	113	0.0030	CC	38	53	0.0014
		T	22	7		CT	22	7	
	LG02_1846779	C	55	59	0.2890	CC	5	11	0.2076
		T	63	51		CT	45	37	
						TT	9	7	
	LG02_1846907	T	70	67	0.6956	CC	10	7	0.4322
		C	50	53		CT	50	53	
\	LG02_1915607	A	69	63	0.4363	AA	18	12	0.4274
		G	51	57		AG	33	39	
						GG	9	9	
	LG02_1919581	T	101	113	0.0127	TT	41	54	0.0042
		C	19	7		TC	19	5	
						CC	0	1	

	LG02_1919619	A	117	117	1	AA	57	57	1
		G	3	3		AG	3	3	
	LG02_1926647	G	101	113	0.0127	GG	42	54	0.0179
		A	19	7		GA	17	5	
						AA	1	1	
pcolce	LG02_2108956	T	68	66	0.7949	TT	17	13	0.5300
		G	52	54		TG	34	40	
						GG	9	7	
prc1	LG02_2223260	T	61	62	0.8972	TT	8	7	0.7801
		G	59	58		TG	45	48	
						GG	7	5	
	LG02_2223450	A	81	71	0.1804	AA	22	14	0.1991
		G	39	49		AG	37	43	
						GG	1	3	
	LG02_2252014	T	68	64	0.6038	TT	17	11	0.3046
		C	52	56		TC	34	42	
						CC	9	7	
pls3	LG02_2307453	A	97	102	0.3911	GG	39	43	0.6752
		G	23	18		GA	19	16	
						AA	2	1	
zmat2	LG02_2485348	C	72	76	0.8409	CC	25	20	0.0277
		A	44	44		CA	22	36	
						AA	11	4	
adamtsl5	LG02_2600869	T	77	66	0.1479	CC	23	14	0.2034
		C	43	54		CT	31	38	
						TT	6	8	
adamts10	LG02_2665634	C	99	112	0.0100	CC	39	53	0.0032
		T	21	8		CT	21	6	
						TT	0	1	

Note：The SNPs marked in red indicate those that have been verified to be significantly related to growth.

Table S4 Quality statistics of RNA-seq data for the eight muscle tissue samples.

Sample ID	Obtained Reads	OBtained Base(Gb)	Q20(%)	Q30(%)	GC(%)	Mapping rate(%)
FG_1	22,301,837	6.57	97.73	93.87	50.76	86.13
FG_2	25,524,419	7.55	98.07	94.83	50.51	82.24
FG_3	20,810,757	5.99	97.70	93.93	50.44	85.85
FG_4	22,806,806	6.70	97.78	93.95	50.19	84.81
SG_1	21,174,346	6.26	97.67	93.59	50.66	87.61
SG_2	23,580,241	6.91	97.97	94.41	50.69	85.17

SG_3	21,852,236	6.42	97.51	93.12	50.87	85.14
SG_4	22,737,869	6.71	97.91	94.27	50.97	83.62

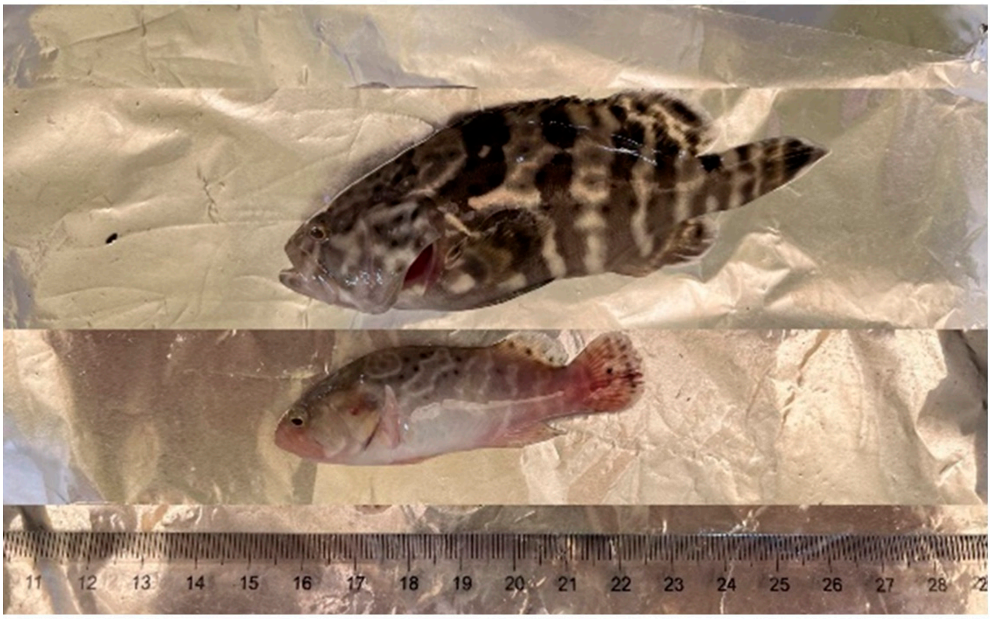

Supplementary Figure S1 Representative appearance of self-cross offspring of hulong hybrid grouper (*E. fuscoguttatus* ♀ × *E. lanceolatus* ♂). The top panel shows a fast-growth individual, while the bottom panel shows a slow-growth individual.

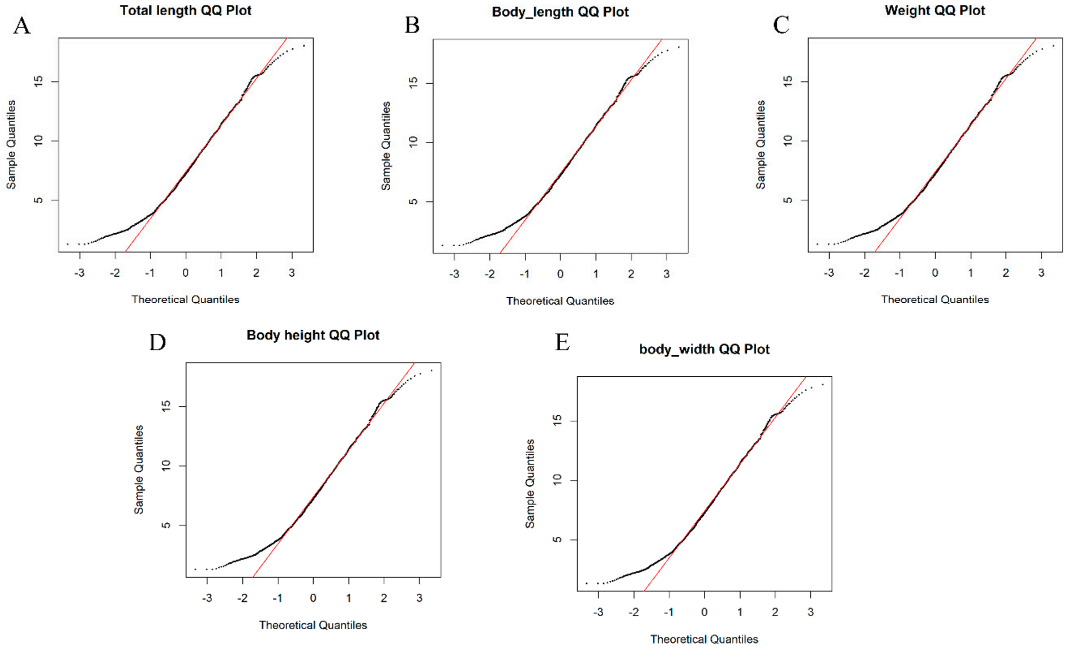

Figure S2 Quantile-Quantile (QQ) plots for assessing the normality of distribution for five growth-related traits. The plots correspond to (A) Total length, (B) Body length, (C) Body weight, (D) Body height, and (E) Body width

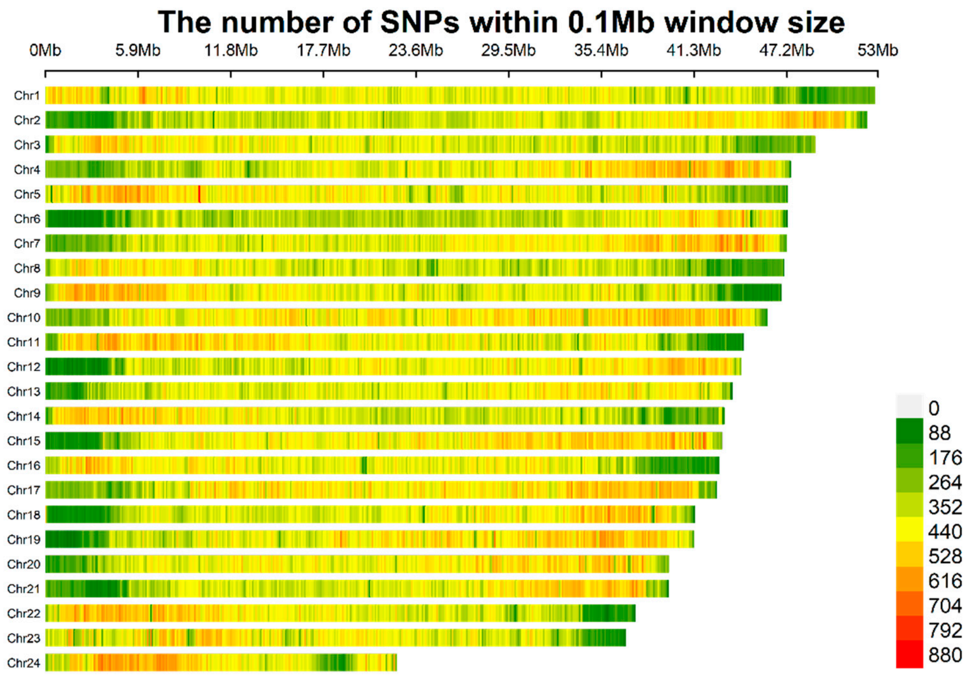

Figure S3 Distribution of SNPs in chromosomes of self-cross population of hulong hybrid grouper

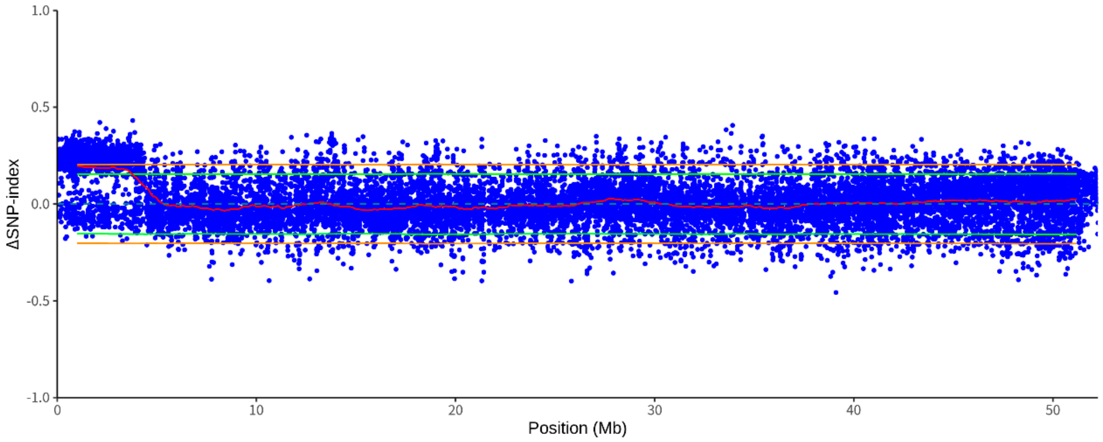

Figure S4 Δ(SNP-index) plot of the candidate region on LG02. The red line is the fitted value. The horizontal lines are the 95% (green) and 99% (orange) confidence thresholds.

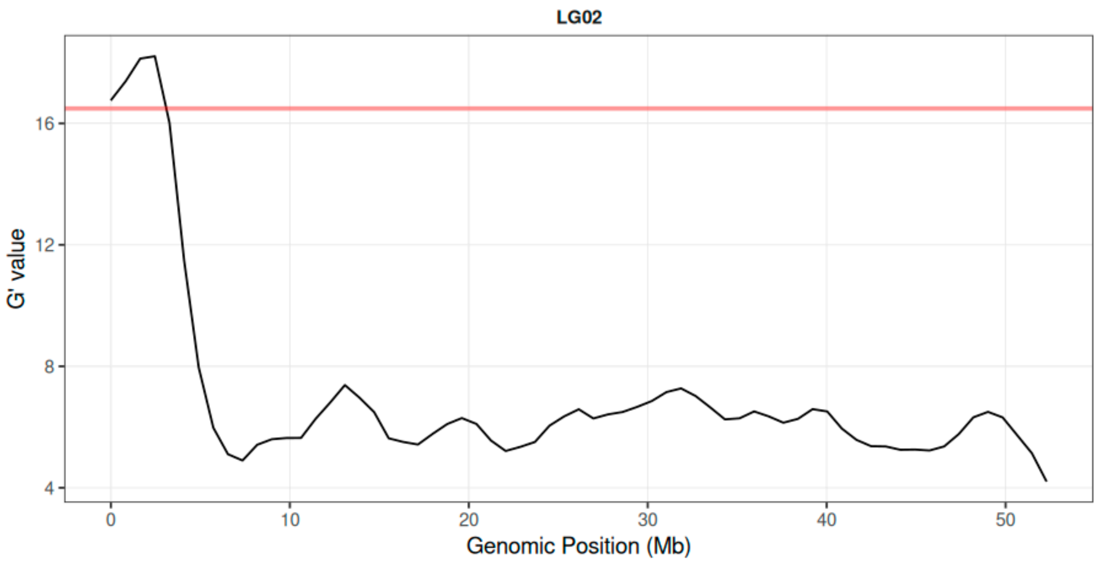

Figure S5 G′ value profile on LG02. The red line shows the significance threshold for $q < 0.05$.
